# Comparative evaluation of microleakage of fifth, sixth, and seventh generation dentin bonding agents: An *in vitro* study

**DOI:** 10.4103/0972-0707.71645

**Published:** 2010

**Authors:** S. Vinay, Vasundhara Shivanna

**Affiliations:** Department of Conservative Dentistry and Endodontics, Bangalore Institute of Dental Sciences and Hospital, Bangalore - 560 029, India; 1 Department of Conservative Dentistry and Endodontics, College of Dental Sciences, Davangere - 577 004, Karnataka, India

**Keywords:** Adper prompt, Clearfil S3 and G-Bond, i-Bond, microleakage, single-bond

## Abstract

**Objectives:**

The purpose of this study was to determine the microleakage in the 5^th^ and 6^th^ generation bonding agents with that compared with the newly introduced 7^th^ generation bonding agents using three bonding agents.

**Materials and Methods:**

A total of 50 recently extracted human upper premolars were subjected to the study. Class V cavity preparations were prepared on the buccal and lingual surfaces of the extracted premolars with occlusal margins in the enamel and gingival margins in the cementum/dentin. The teeth were divided into five groups of 10 teeth each and 20 cavity preparations per group. In the experimental groups, cavities were treated with Single-Bond, Adper Prompt, i-Bond, Clearfil S3, and G-Bond as the dentin bonding agents. After the application of the dentin bonding agents, the cavity preparations were restored with resin composite (Clearfil APX). The specimens were thermocycled, stained with methylene blue dye, and sectioned to evaluate the dye penetration. Data were analyzed using the one-way ANOVA test (Kruskal–Wallis) and Dunn’s procedure for pairwise comparison of the data.

**Results:**

This study showed that at the coronal margin and the apical margins, the preparations treated with Clearfil S3 showed significantly less leakage than the other groups. Enamel margins provided better marginal sealing than dentin/ cementum margins.

**Interpretation and Conclusion:**

The study demonstrated that Clearfil S3 bond had a better sealing ability at both coronal (enamel) and apical (dentin/cementum) margins compared with the other dentin bonding agents used.

## INTRODUCTION

Vital dentin is an extension of certain pulpal tissues presenting the first line of defense against patient hypersensitivity and long-term bacterial microleakage that can lead to recurrent caries and pulpal irritation. The most significant factor in determining resistance to recurrent caries, postoperative sensitivity, marginal staining, and pulpal damage is the ability of the restorative material/ adhesive to seal the restorative interface with the adjacent tooth surface.[1,2]

The most current breakthrough in dental adhesive systems used in the composite bonding restorations is the 7^th^ generation bonding systems. The claim of the manufacturer is that this bonding system will eliminate mixing and etching, while, at the same time, accomplishing the priming and the bonding of the dental surfaces.[3] This advancement drastically reduced the microleakage between the tooth–resin interfaces when compared with the older generation of composites, but is still significant in terms of its dental use. It is well documented that the setting reaction of the resin composites may lead to the formation of a contraction gap at the tooth restoration interface.[4] This gap results in the passage of bacteria, fluids, or ions between the cavity wall and the resin composite, a process defined as microleakage.[3,5]

## MATERIALS AND METHODS

Fifty recently extracted, noncarious, unrestored human premolars (indicated for extraction due to orthodontic reasons) were collected from the Department of Oral Surgery. The teeth were scraped of any residual tissue tags, kept in 2.6% sodium hypochlorite solution, and rinsed under running water for 15 min each.

Class V cavity preparations were prepared on the facial and lingual surfaces of each tooth with a cylindrical diamond bur. No bevels were used in the preparation. Each cavity was approximately 3-mm-wide, mesio-distally paralleling the Cemento-enamel junction (CEJ), 2-mm-wide occlusogingivally and 1.5-mm-deep approximately. The gingival half of the preparation was extended 1 mm below the CEJ.

Group I: Ten teeth (20 cavity preparations) used the 5th generation bonding agent SINGLE BOND (3M ESPE-3M General Offices St. Paul, MN 55144 USA).

Group II: Ten teeth (20 cavity preparations) used the 6th generation bonding agent ADPER PROMPT (3M ESPE).

Group III: Ten teeth (20 cavity preparations) used the 7th generation bonding agent i-BOND (Heraeus Kulzer D-63450, HANAU, GERMANY).

Group IV: Ten teeth (20 cavity preparations) used the 7th generation bonding agent CLEARFIL S3 (Kuraray Medical Inc- 1621 Sakazu, Kurashiki, Okayama 710-0801, Japan).

Group V: Ten teeth (20 cavity preparations) used the 7th generation bonding agent G-BOND (GC CORPORATION:76-1 Hasunuma-cho, Itabashi-ku, Tokyo 174-8585 Japan).

The dentin bonding systems were applied following the manufacturer’s instructions and the cavities were restored with resin composite (Clearfil APX). The cavosurface margins were then finished with a finishing bur and sof-lex discs.

All the teeth were subjected to 1,000 thermal cycles between water baths of 5±2°C and 55±2°C, with a dwell time of 30 s.

The specimens were subjected to dye-leakage tests. The teeth were covered with two coats of nail varnish to within 1 mm of the tooth-restoration margin after the root apices were sealed with modelling wax. The specimens were immersed in methylene blue solution for 24 h at 37°C.

After staining, the teeth were rinsed to remove residual stain and the radicular parts of the teeth were cut 4–5 mm below the CEJ. The coronal parts were sectioned mesiodistally and then buccolingually in the approximate center of the restoration with a low-speed diamond saw. Microleakage was assessed for both occlusal (enamel) and gingival (dentin/cementum) margins using a stereomicroscope at original magnification (×16).

The depth of the stain (dye leakage) was judged according to the following scale:

0 – No leakage
1 – Dye penetration up to one-third of the cavity preparation.
2 – Dye penetration up to two-third of the cavity preparation.
3 – Dye penetration up to full-cavity depth.
4 – Dye penetration onto the axial wall of the cavity preparation.

A nonparametric analysis of variance (ANOVA) test (Kruskal–Wallis) was used to determine whether there were significant differences among the groups. Pairwise comparisons between groups were made using Dunn’s procedure for nonparametric data. Occlusal and gingival margins within the treatment groups were compared using the Wilcoxon signed rank test. The level of significance was established as *P* <0.05 for all tests.

## RESULTS

Table 1 shows the distribution of microleakage scores at the coronal margins (enamel) for all the groups.

**Table 1:** Distribution of microleakage scores at the coronal margins (Enamel)

Groups	Score					Mean	S. D.	% of no microleakage
	0	1	2	3	4			
Single Bond	25	15	0	0	0	0.38	0.490	62.5
Adper Prompt	6	22	10	2	0	1.2	0.758	15
I-Bond	10	25	5	0	0	0.88	0.607	25
Clearfil S3	26	14	0	0	0	0.35	0.483	65
G-Bond	22	12	4	2	0	0.65	0.864	55

Table 2 shows the distribution of microleakage scores at the apical margins (cementum) for all the groups.

**Table 2:** Distribution of microleakage scores at the apical margins (Cementum)

Groups	Score					Mean	S. D.	% of no microleakage
	0	1	2	3	4			
Single Bond	1	15	17	7	0	1.75	0.776	2.5
Adper Prompt	0	7	22	10	1	2.125	0.723	0
I-Bond	1	16	20	1	2	1.68	0.797	2.5
Clearfil S3	10	14	10	4	2	1.35	1.122	25
G-Bond	9	14	10	3	4	1.48	1.219	22.5

Table 3 shows a comparison between microleakage scores in the enamel and cementum.

**Table 3:** Comparison between microleakage scores in the enamel and cementum

Groups	Z-Value	*P*-value	Significance
Single Bond	5.456	<0.001	HS
Adper Prompt	4.524	<0.001	HS
I-Bond	4.264	<0.001	HS
Clearfil S3	4.358	<0.001	HS
G-Bond	3.283	<0.01	VS

In all the groups (Groups I, II, III, and IV), the difference in microleakage scores in the enamel and cementum margins is highly significant (*P* < 0.001), whereas in Group V, the difference was very significant (*P* < 0.01) between microleakage scores in the enamel and cementum margins.

### Summary of the results

At the coronal (enamel) margins of the cavity, Group IV (Clearfil S3) exhibited less microleakage than the other bonding systems. At the apical (dentin/cementum) margins of the cavity, again, Group IV (Clearfil S3) exhibited less microleakage than the other bonding systems.

## DISCUSSION

The concept of bonding-restorative materials to tooth structures was first seriously considered nearly 5 decades ago, when Dr. Gunnar Ryge of Marquette University and Professor Philip discussed the theoretical possibility of bonding resins to dentin. This was long before the advent of composites. While such an objective was highly desirable, at that time, it seemed to be only a remote possibility.[6] Major drawbacks of this approach are that adhesive techniques still accompany a higher placement complexity and technique sensitivity.[7]

Although enamel adhesion is a predictable and established entity, an adequate bond to dentin is more difficult to achieve. To overcome this challenge, technological advancements of dentin adhesives have, at this time, evolved into two trends: total acid-etching techniques [5^th^ generation Dentin bonding agents (DBA)] and self-etching primer technique (6^th^ and 7^th^ generation DBA).[3]

Self-etch adhesives, composed of aqueous mixture of acidic functional monomers that are generally phosphoric acid esters, do not require a separate acid-etch component and subsequent rinsing procedures. Self-etch systems also purportedly enhance marginal integrity and reduce or eliminate patient symptoms.[7]

The results showed that at the coronal (enamel) margins of the cavity, Clearfil S3 exhibited less microleakage than the other bonding systems, followed closely by Single- Bond and G-bond. The i-Bond and Adper Prompt showed significantly greater microleakage at the coronal margins and Adper Prompt showed the highest microleakage among all the adhesive systems that were compared.

The probable explanations for these results of Clearfil S3 include:[8,9]

- The type of solvent used in the adhesive systems. Instead of acetone, Clearfil S3 Bond uses alcohol as the primer component solvent. It has been proposed that acetone-based adhesives require the use of a wet bonding technique with a relatively small window of opportunity to achieve optimal hybridization.
- The Clearfil S3 Bond formulation includes a proprietary “Molecular Dispersion Technology,” enabling a twophase liquid, hydrophilic/hydrophobic component homogenous state at the molecular level, reportedly resulting in reduction and/or loss of water droplets at the adhesive interface and, therein, a superior bond.
- Also, the 10-methacryloyloxydecyl dihydrogen phosphate adhesive monomer molecular structure allows for decalcification and penetration into the tooth structure, creating a chemical bond to calcium.

There was a significant difference in the microleakage between Single Bond with Adper Prompt and i-Bond.[10]

The probable explanations for these results of Single Bond include:

1. Single bond combines the functions of the primer and adhesive components of the conventional three-step adhesive system and has alcohol and water as solvents in its composition.
2. The presence of alcohol in the adhesive increases the diffusion into the dentin, thereby enhancing the adhesion. The moisture of dentin tubules pulls the alcohol into and in between the tubules, taking the resin with it. The alcohol and moisture then vaporize from the substrate leaving behind the resin.[11]

There was significant difference in the microleakage between G-Bond when compared with Adper Prompt and i-Bond.[12]

The probable explanations for these results of G-Bond are:

- The interface formed is totally different from that of the interface formed by earlier bonding materials. The surface of the dentin is decalcified only slightly and there is almost no exposure of the collagen fibers. This suggests that an extremely thin (300 nm or less) interface is formed and that in this area, functional monomers contained in the bonding material react with hydroxyapatite at the “nano” level to form insoluble calcium. Therefore, the interface formed by G-Bond is expected to be stronger and more durable than that formed by other bonding materials.
- It would appear appropriate to call the interface exhibiting this a nano interaction zone, or a reacted layer at the “nano” level, as opposed to the traditional hybrid layer appellation.
- G-Bond’s 5% filler further seals the tubules and decreases pulpal sensitivity. There are fewer voids between the thin adhesive layer and the dentin, which minimizes microleakage and prevents microbial invasion.

The results showed that i-Bond showed significantly greater microleakage at the coronal margins when compared with ClearfilS3, G-Bond and Single-Bond and less microleakage than Adper Prompt.[8,13]

The probable explanations for these results of i-Bond include:

1. The technique protocols (drying procedures) associated with i-Bond proved to be labor-intensive and may not be realistic for some practitioners in a clinical setting.
2. Possible explanations for this occurrence include multiple applications (manufacturer’s instructions) of the i-Bond adhesive onto the preparation surfaces and increased waiting periods prior to light polymerization.

The most extensive microleakage of the dye was seen with Adper Prompt as compared with all the other adhesives.[14]

The probable explanations for these results of Adper Prompt include:

1. It is claimed that the absence of the fillers in the 6^th^ generation dentin bonding agents can explain the poor results in terms of microleakage.
2. It was also stated that there is a possibility that the lack of a separate primer may reduce the infiltration depth or the wettability of the dentin adhesives, thereby reducing the sealing adhesion and the sealing capacity.

None of the groups of the dentin bonding agents prevented the microleakage at the restoration–tooth interface.[3,8,15]

The results also showed that at the apical (dentin/cementum) margins of the cavity, again, Clearfil S3 exhibited less microleakage than the other bonding systems followed closely by G-Bond. The i-Bond, Single Bond, and Adper Prompt showed significantly more microleakage at the apical margins, and Adper Prompt showed the highest microleakage among all the adhesive systems that were compared.

The results also showed that the microleakage at the enamel margins is less compared to that of the microleakage at the dentin/cementum margins.[1,16]

These results were in accordance with the previous results.[1,7]

The most probable reasons for the less microleakage at the enamel margins compared to that of the microleakage at the dentin/cementum margins are:

1. The hypermineralization of the dentin surface and subsequent collapse of the collagen fibrillar network, cavity configuration (C Factor), dentin tubule orientation to the cervical wall (CEJ), organic content of dentin substrate and movement of dentinal tubular fluids, incomplete alteration/removal of the smear layer by acidic primers for adequate demineralization and hybrid layer formation, and inefficient infiltration/ penetration of primer components into the demineralized collagen fibrillar network. Solvent carriers (water, alcohol, and acetone) in the adhesive agent reacted differently with varying degrees of surface moisture present in the tubules.[8]
2. In class V cavities, located at approximately 1 mm from the CEJ, dentinal tubules are oriented parallel to the cervical wall. A classic hybrid layer formation is absent in the dentinal margins, and this absence is an important cause of leakage.[1]

Microleakage tests provide adequate screening methods, possibly determining clinical success and longevity of the adhesive systems. Although this study was conducted *in vitro*, this can be a screening apparatus for ensuing *in vivo* studies. Previous research indicates that data obtained from *in vitro* microleakage testing may be useful but not always necessarily reproducible in clinical *in vivo* settings.[5]

The results of this study demonstrate that the dynamic nature of the dentin substrate morphology is indeed an important factor and, possibly, an insurmountable impediment for perfect adhesion of restorative materials to the tooth structure. Clinical trials should be performed for carious and noncarious Class V cervical lesions to assess the performance of these new adhesive systems before definite conclusions can be formulated.
